# Treatment of Cholera Infantum

**Published:** 1876-11

**Authors:** Edward Waldo Emmerson


### TREATMENT OF CHOLERA INFANTUM.

By EDWARD WALDO EMMERSON, M.D.

Waiving the question of prophylaxis and its ordinary, and its corollary, the question how to directly destroy or neutralize the organic irritant (if such exist) after its introduction into the body, the first indication is to correct the dangerous and unfair distribution of the blood in the body, to which the purging, vomiting, cramps, and coldness, seem to be directly due, and later the greater danger of coma, convulsions, or paralysis of the heart.

Second. If we fail in the first attempt, or do not succeed until late, we should supply the water and perhaps also the salts drained from the blood, as the thickening of the blood would prevent the good effects of the natural turn of the disease, should we have to wait for that, and perhaps dispose to various organic lesions.

Third. We should attend to the general hygiene, diet, etc., of the patients.

As to the first indication, the problem is how to cause dilatation of the peripheral vessels and contraction of the overloaded abdominal ones. If we had any means of getting directly at the splanchnic nerves, we could probably by galvanization of them directly cause the contraction of mesenteric vessels. Ludwig and Thiry found that after section the spinal cord in the neck, whereby dilatation of the mesenteric vessels was caused, galvanization of the lower segment would cause extreme contraction of them. Possibly galvanization applied over the middle dorsal region of a patient might produce the same effect. Chapman maintains that he can occasion it by ice bags applied to the spine, which he uses to check diarrhœas and reflex vomiting.

Bruckner, a German writer, claims that cold sand bags of moderate weight, laid on the abdomen of cholera patients, mechanically check the access of blood to the abdominal vessels and favor its escape. Transudation is thus hindered, and perhaps absorption is favored; moreover, the peristaltic movements of the bowels are not so free. These sand bags might be used carefully, with hot applications to the extremities.

We have a much better chance of succcss, however, if we try to unload the abdominal vessels by relaxing the peripheral ones by means of strong derivatives applied to the surface. Steiner strongly urges baths of from 99° to 104° Fahr. in the algid stage, combined with stimulants internally, and Leube, in Ziemssen's Cyclopædia, recommends the same. The distinction, too often neglected, between a warm bath and a hot bath is of vital importance here. No bath of less than 99° would be desirable. A writer in an English journal within a year or two, whose name I have lost, mentions his very gratifying success in treating the algid stage of Asiatic cholera by prolonged hot mustard packs. In accordance with this plan I treated three cholera infantum patients last summer, who were rapidly cooling off and assuming the characteristic pinched appearances of collapse, by suddenly wrapping them to the chin in cloths wrung out in hot water and mustard, with a blanket outside, and while thus mummied feeding them with plenty of ice-water and a little brandy. The pack was kept up half an hour or more, and during that time the change in the child's appearance was remarkable; the color and warmth returned to the surface, the tissues filled out, the features lost the pinched and old look, a natural perspiration broke out, the vomiting ceased, and the discharges grew less frequent. The mustard sheet was then withdrawn, but the child left enveloped closely in the warm, moist blanket. The pack in one instance had to be renewed at intervals, as a tendency to relapse manifested itself after some hours, but the condition of all mended in marked manner after the first application, and all made a good recovery.

With regard to medication, if the choleraic state last any length of time, the blood must necessarily be altered by its drain of water and salts. Water, then, is the first medicine indicated, and should be constantly given in the form of ice-pills or spoonfuls of ice water. Small enemata of slightly salt water immediately after a dejection might help to supply the lost fluid. Should vomiting and purging go far enough to cause a fear that the blood was becoming too much thickened, intra-venous injections of water should be tried, and if it were thrown in at a temperature of 100° the heat might help relax the surface vessels. Milk and blood have also been used, but water seems more indicated, as in this disease the blood loses little albumen and no corpuscles.

As to the administration of drugs by the mouth, the fact of the probable very slight power of absorption at that time is usually overlooked. It is found that belladonna introduced into the stomach in large doses will not dilate the pupils. The medicines, stimulants, and food, then, can have little power in the present condition, nor yet help to bring on reaction, and if often repeated they may, when reaction sets in, be all greedily absorbed at once, and so do great harm, a fact to which Meigs and Pepper very properly call attention with regard to pouring in opium and alchohol in the algid stage. Internal administration of antiseptics has not so far semed to fulfill the expectations of its advocates. As for calomel, it seems hardly indicated in the pure choleraic stage, unless there is the best reason to believe that some crude ingesta still present in the intestine demand a cathartic.

In the *Practitioner* of July, 1875, was a very striking article on the use of subcutaneous injections of chloral in the evacuant or algid stage of cholera, by Surgeon A. R. Hall, with accounts of cases treated by him and Mr. Higginson, another English army surgeon. The number of cases treated by these two gentlemen were large, and the onset severe and alarming, but they lost hardly a case. They injected two-grain doses of chloral, diluted with ten times as much water, into the arms and legs of patients, some in extreme collapse, and in almost every case good and speedy recovery ensued. Few patients had more than eight to ten grains in all. Mr. Hall's theory was that the vascular condition was due to extreme vaso-motor irritation, and that the usual stimulant treatment only heightened the difficulty, as was shown by its small percentage of recoveries, sometimes only eighteen per cent. So he looked about for a sedative to relax the general spasm, and tried chloral with the brilliant results above mentioned. It is interesting to know that the government in India have taken pains to publish and circulate Mr. Hall's happy experience in the treatment of cholera collapse. His method seems to be well vouched for, and I see no reason why it should not be applicable to the choleraic state in children, if the injections were given progressively and carefully watched.

One word, in conclusion, as to babies' food, though that subject has been so well treated at recent meetings of the society that it is almost superfluous to say a word more. There is a point which I wish to allude to, namely, the great popularity among the rich and poor of the nursing bottle with the flexible tube. It is an invention of which Herod might have been proud. It is always in the baby wagon or the crib, in hot sun or close air. The child falls asleep with its nipple in his mouth. The mouth is usually never washed; the bottle and tube are, "with scalding water and with soda," so the mother says if you ask. Smell it, and see what you think. Take a parallel case. What prospect could a man have of immediate and satisfactory recovery from cholera morbus, or even dyspepsia, who should eat soup, freshly made perhaps, but out of a tureen which had been standing half a day with the remains of yesterday's soup in it, in a close room with a temperature of 90°; who, moreover, should never rinse out his mouth nor allow time for digestion, but should go to sleep with a piece of bread soaked in soup in his mouth, and if colic or oppression caused him to complain or waking, should at once take more soup out of the unscalded tureen? This is not an agreeable picture, but it is a fair analogy. Is a teething baby's stomach stronger than a man's, that the doctor should tolerate the form of nursing bottle which encourages and contemplates a management of his diet exactly parallel to that in the unattractive picture I have just drawn?—*Boston Medical and Surgical Journal.*

